# Direct risk standardisation: a new method for comparing casemix adjusted event rates using complex models

**DOI:** 10.1186/1471-2288-13-133

**Published:** 2013-10-29

**Authors:** Jon Nicholl, Richard M Jacques, Michael J Campbell

**Affiliations:** 1 School of Health and Related Research (ScHARR), University of Sheffield, 30 Regent Street, Sheffield, S1 4DA, UK

**Keywords:** Standardisation, Standardised mortality ratio, Hospital performance, Logistic regression models, Casemix adjustment

## Abstract

**Background:**

Comparison of outcomes between populations or centres may be confounded by any casemix differences and standardisation is carried out to avoid this. However, when the casemix adjustment models are large and complex, direct standardisation has been described as “practically impossible”, and indirect standardisation may lead to unfair comparisons. We propose a new method of directly standardising for risk rather than standardising for casemix which overcomes these problems.

**Methods:**

Using a casemix model which is the same model as would be used in indirect standardisation, the risk in individuals is estimated. Risk categories are defined, and event rates in each category for each centre to be compared are calculated. A weighted sum of the risk category specific event rates is then calculated. We have illustrated this method using data on 6 million admissions to 146 hospitals in England in 2007/8 and an existing model with over 5000 casemix combinations, and a second dataset of 18,668 adult emergency admissions to 9 centres in the UK and overseas and a published model with over 20,000 casemix combinations and a continuous covariate.

**Results:**

Substantial differences between conventional directly casemix standardised rates and rates from direct risk standardisation (DRS) were found. Results based on DRS were very similar to Standardised Mortality Ratios (SMRs) obtained from indirect standardisation, with similar standard errors.

**Conclusions:**

Direct risk standardisation using our proposed method is as straightforward as using conventional direct or indirect standardisation, always enables fair comparisons of performance to be made, can use continuous casemix covariates, and was found in our examples to have similar standard errors to the SMR. It should be preferred when there is a risk that conventional direct or indirect standardisation will lead to unfair comparisons.

## Background

In all branches of the health and social sciences, and especially in public health and health services research, we need to be able to compare outcomes of groups of patients or people with different exposures in order to understand the impact of the exposures. These exposures include different interventions and services, as well as different environments.

Comparison of outcomes can be difficult because of differences in the characteristics of the patients or populations being exposed in different ways. The distribution of these characteristics is known as the casemix, and when the casemix is associated with the outcomes, comparisons of outcomes are confounded by any differences in casemix. In this case comparisons are sometimes made by calculating a measure of the event rate in each exposure group being compared which is standardised for casemix. When the number of groups being compared is not too large this can be done by including a term for the effect of each exposure in the casemix adjustment model. However, when many groups are being compared this may not be possible and standardisation is carried out. There are numerous methods that can be used to standardise for casemix [1] but the most frequently used are direct and indirect standardisation [2,3].

First, we rehearse some well-known problems with both direct and indirect standardisation, and then we propose a new approach which overcomes the problem with direct standardisation. We have termed this new approach Direct Risk Standardisation (DRS). To discuss and illustrate these issues, we have used the example of comparing hospital mortality, and throughout this paper we refer to the populations or exposure groups being compared as 'centres’, and people as 'patients’, but the methods are quite general.

### Direct standardisation

In direct standardisation, for each centre event rates are calculated for every combination of the casemix variables and then these casemix specific event rates are combined using a set of weights which is the same for all the centres. One problem with this method is that it can’t be used when any of the casemix variables are continuous unless they are first grouped into categories. A second more serious problem with direct standardisation is that some casemix combinations in some centres may have no patients or people. This may be for a structural reason (eg gynaecological conditions in men), an organisational reason (e.g. the hospital doesn’t treat children) or a random reason (eg for some uncommon conditions there may be no cases in some hospitals in some years). Directly standardised comparisons between centres with different numbers or patterns of empty casemix cells (i.e. due to random or organisational rather than structural reasons) are no longer fair [4]. For example, suppose there are just three casemix groups (eg children, adults, elderly) and two hospitals being compared, one of which (hospital 2) admits no children and for similar patients treated by both hospitals is 20% *worse* than the other, as illustrated in Table 1.

**Table 1:** Example of effect on direct stanardisation of missing casemix combinations

	**Age specific death rate per 100 admissions**			**Directly standardised rates**
	**Children**	**Adults**	**Elderly**	
**Hospital 1**	20	10	20	(0.25×20) + (0.5×10) + (0.25×20) = 15
**Hospital 2**		12	24	(0.5×12) + (0.25×24) = 12

Now suppose the weights used to combine the hospital mortality rates are the national proportions of child, adult, and elderly patients which are 25%, 50%, and 25% say. Then the directly standardised rates show that Hospital 2 is 20% *better* than Hospital 1. This has arisen because the total of the effective weights used for each hospital are different.

A partial solution to this problem is to recalculate the weights for each centre so that they always sum to 1.0. In the example in Table 1 the weights used for hospital 2 only sum to 0.75. Dividing the weights for Hospital 2 by 0.75 so that the weights used again sum to 1.0 makes the directly standardised rate in Hospital 2 equal to 16 deaths per 100 admissions indicating that hospital 2 is about 7% worse than hospital 1. As this example illustrates, recalculating the weights won’t completely resolve the problem if the missing weights apply to cells which have high or low event rates, and the method would still have the disadvantage of not being usable with continuous covariates.

### Indirect standardisation

In indirect standardisation a set of standard casemix specific event rates is 'weighted’ by the local population casemix. In effect this calculates the number of events expected in the local population if the standard event rates had happened. The indirectly standardised rate is usually presented as the ratio of the observed number of events to the expected number. When the events are deaths this is known as the Standardised Mortality Ratio (SMR) and we use this term for all standardised event ratios.

A simple way to calculate an SMR is to use a logistic regression model with the casemix as covariates to estimate the probability of death in each patient across all comparators together. These probabilities are summed over all the patients in each comparator to derive the expected number of deaths in that comparator. The two methods - using locally weighted standard casemix specific event rates or logistic regression - will give the same results if the standard casemix specific event rates are derived from the pooled data and the logistic regression includes all possible interactions rather than just main effects say [5]. However, the logistic regression method has the advantages of being able to use continuous covariates and being able to simplify large complex casemix models.

The problems of indirect standardisation have previously been reported [2,6]– [10]. Briefly, since the set of weights reflects the local population casemix they are different for each centre and so the SMRs aren’t strictly comparable between centres [8]. When the casemix is very different the SMRs may not be comparable at all [7].

The problem of non-comparability is illustrated in Table 2 which shows a simple example with two hospitals with identical casemix specific mortality rates but different casemix. Though the performance of the two hospitals is identical, the hospital with the largest proportion of high risk patients (40% vs 30%) has a lower SMR (105 vs 112).

**Table 2:** **Example of non**-**comparability of SMRs**

**Casemix group**	**National standard death rate**	**Hospital A: Death rate**	**Hospital A: Casemix proportions**	**Hospital B: Death rate**	**Hospital B: Casemix proportions**
**1. High risk**	0.9	0.8	0.4	0.8	0.3
**2. Low risk**	0.1	0.2	0.6	0.2	0.7
**SMR**			O = (0.4×0.8) + (0.6×0.2)		O = (0.3×0.8) + (0.7×0.2)
			E = (0.4×0.9) + (0.6×0.1)		E = (0.3×0.9) + (0.7×0.1)
			SMR = 105		SMR = 112

Thus standardisation for casemix indirectly via SMRs cannot yield fair comparisons, but the correct way (direct standardisation) also may not work because of different patterns of empty casemix combinations and is not possible with continuous covariates.

This paper explores an alternative solution to the calculation of comparable standardised rates.

## Methods

### Calculate event rates in risk groups rather than casemix groups

One possible approach to calculating directly standardised rates when there are empty casemix combinations is to differentiate between casemix standardisation and risk adjustment. The reason for the non-comparability of crude event rates is usually said to be because of differences in casemix in the comparators. However, non-comparability actually follows from differences in the risk distribution in the comparators. If different casemixes gave the same risk distribution, crude unadjusted comparisons would still be fair. For example, if older patients admitted to hospital for elective procedures have the same risk of mortality as younger emergency patients, then unadjusted comparisons of mortality between two hospitals one of which had a majority of older elective patients and the other a majority of younger emergencies could still be fair.

It follows from this that a solution to the difficulty of calculating directly standardised rates in the presence of empty casemix combinations is to convert the complex multidimensional casemix to a simple one-dimensional risk distribution and then directly standardise across the risk distribution. The risk is calculated using a standard logistic regression modelling approach using the casemix variables. This model is the same as would be used in indirect standardisation and can use continuous covariates as well as fixed factors. The model is fitted to the whole dataset aggregated across the centres (eg the institutions, populations, or years) to be compared. Estimates of the predicted risk for each case in the aggregated dataset are obtained and using this each person is assigned to a risk category. Observed event rates within each risk category are then calculated for each centre, and these event rates are weighted and combined using a standard set of weights. In order to make comparisons easy, an index similar to the Standardised Mortality Ratio (SMR), the Comparative Mortality Figure (CMF) can be calculated by dividing the standardised rate by the overall rate (see the Appendix in Additional file 1) [3].

### Calculating directly risk standardised rates

The first step is to estimate the risk of an event for each person in the whole population (ie aggregated across all comparators) which is usually done using a logistic regression model. How should this model be specified? This is the same problem for all methods of standardisation. In conventional direct casemix standardisation, the casemix variables must be chosen and any continuous covariates have to be converted into categorical factors. For indirect standardisation a logistic regression model using the casemix variables has to be specified in order to estimate the expected numbers of events from the predicted risks. Misspecification of the model is likely to lead to invalid comparisons between centres for all methods of standardisation, including our proposed DRS method. However, for the purposes of this study, which compares different methods of standardisation rather than different models for standardisation, we have simply used the same models for each of the methods in order to ensure comparability.

The second step is to assign each case to a risk category. These risk categories are defined using the whole aggregated dataset. There are several options for defining the risk categories and choosing the weights for standardisation (see Table 3). It is important to use risk categories which don’t mean that some centres have risk categories with no patients in them since this obviates the point of the proposed risk adjustment method. For example, choosing risk categories of equal width (such as a risk from 0.0–0.1, 0.1–0.2, 0.2-0.3, etc.) may mean that there are no patients in some of the lowest or highest risk groups in some centres, and this method will not usually work. Choosing groups with equal numbers of patients in each group will mean that there are no events in some risk categories if the risk over the whole population is small, such as with in-hospital mortality which is typically about 5%. Choosing groups with equal numbers of observed or predicted events will usually ensure that there are some events and therefore some patients in each risk category in every centre unless there are some centres with very few cases.

**Table 3:** Methods of calculating risk categories and weights

**Creating categories of risk**	**Weights for combining risk category specific event rates for each centre**
Equal width: 0.0-0.1, 0.1-0.2, 0.2-0.3, etc.	Equal
Equal numbers of patients in each	Proportion of all patients in each category
Equal numbers of observed deaths in each	Proportion of all observed deaths in each category
Equal numbers of predicted deaths in each	Proportion of all predicted deaths in each category

We found that choosing categories with equal numbers of observed events is simpler than choosing categories with equal numbers of predicted events and gives similar results, so this is our preferred method. Of course all patients with the same casemix fall into the same risk category, and so it may not be possible to create categories with exactly the same number of events, rather this is a guiding principle for choosing categories.

The number of categories to use is also a matter of choice. The method only works exactly (ie centres with identical casemix specific risks have identical DRS rates) if all the patients grouped into the same risk category have the same risk. So the more risk groups that are used the more exact the method becomes. However, the more categories that are used the more chance that there will be some centres with some risk categories with no cases. Our results suggest that about 10 categories should be used if possible (see below), although up to 20 could be used for very large datasets.

With regard to the weights for combining the risk category specific event rates, the natural weights are the proportion of patients in each category in the whole population aggregated across comparators since this means that the standardised event rate for the whole population is the same as the observed event rate. Furthermore, with these weights the CMF for a centre is just the DRS rate for that centre divided by the observed event rate for the whole population in all centres combined.

### Examples

#### Data and methods

We have explored this proposal using two datasets.

First we have used Hospital Episode Statistics (HES) data for approximately 6 million admissions to 146 general and acute NHS hospitals in England during 2007/8 linked to mortality files. The events that we have used are deaths 30 days post admission. The estimation of the probabilities of death has been carried out using the architecture of the standard Summary Hospital Mortality Index (SHMI) model using clinical code on admission, age, sex, mode of admission and co-morbidities (using the Charlson index treated as a categorical variable) [11]. The SHMI model has been fitted to the aggregate data for all hospitals together using standard logistic regression, and the predicted probabilities of 30-day mortality were extracted to estimate risks and calculate expected numbers of events.

The second dataset we have used is the data on 18,668 adult emergency medical admissions from 9 centres in the UK and overseas collected for the DAVROS project which has developed models for casemix adjustment [12]. We have used the model including age, ICD10, and history of malignancy together with categorical values for six physiological measurements. We have used age as a continuous covariate to illustrate the method. Cases with any missing data have been deleted, and this model has been fitted to the aggregate data for all 9 centres using standard logistic regression and the predicted probabilities of 7-day mortality extracted.

We have calculated an SMR for each hospital in the HES data and each centre in the DAVROS data using the ratio of the observed number of deaths to the sum of the predicted probabilities from the models.

In both cases we have calculated the DRS rate using approximately equal numbers of observed deaths in the aggregate data to define 20 risk categories for the HES data and 10 for the DAVROS data. We have weighted the centre-specific mortality rates in the risk categories by the proportion of patients in the risk category in the aggregate data. We have calculated the DRS CMF by dividing the DRS rate by the overall population mortality rate in the aggregate data (that is the total number of deaths divided by the total population).

Standard errors for the SMR and the DRS CMF for the DAVROS data have been calculated using the formulae in the Appendix (see Additional file 1) and by a simple bootstrap taking 1000 samples with replacement from each centre, calculating the SMRs and DRS CMFs for each sample, and calculating their standard deviation from the average value in the bootstrap samples. Standard errors for the estimates for the 146 hospitals in the HES data are not shown.

We have also re-calculated the DRS CMFs for the 146 hospitals in the HES data using between 5 and 25 risk categories in order to examine the reliability of the method with different numbers of categories. We have calculated the rank correlations between the values of DSR CMFs calculated using different numbers of categories.

For the HES data we have calculated the weights that a conventional directly standardised rate (DSR) would use (that is, the proportion of all the patients in the whole dataset falling into each possible casemix combination). In calculating the DSR for a particular hospital, if there are no patients in a casemix combination the weight for that combination is not used. So for each hospital we have summed the weights that have actually been used in calculating the DSR for that hospital. We have not done this for the DAVROS data as age has been treated as a continuous variable and a conventional DSR cannot be calculated.

## Results

### HES data

**Figure 1:**
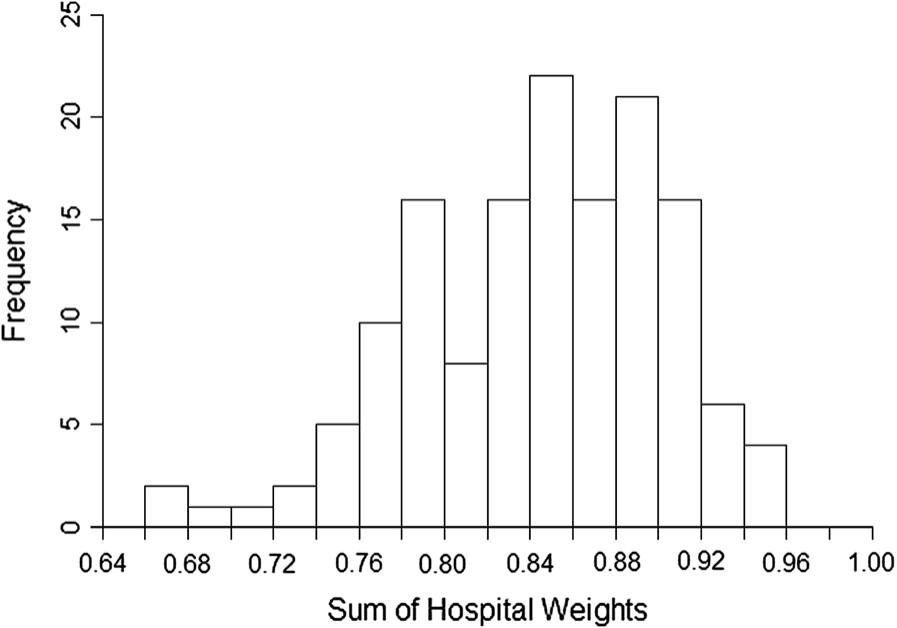
Histogram showing the sum of the effective weights used in the calculation of conventional directly casemix standardised rates in 146 English hospitals.

Figure 1 shows the sum of the weights actually used in the conventional direct casemix standardisation for each of the 146 hospitals. In every hospital there are some casemix combinations with no patients, so the weights actually used do not sum to 1.0 in any hospital and are often less than 0.8. In response to this problem, the directly casemix standardised CMFs used here for comparing with the SMRs and directly risk standardised CMFs have been calculated by adjusting the weights so that they sum to 1.0 in each hospital.

**Figure 2:**
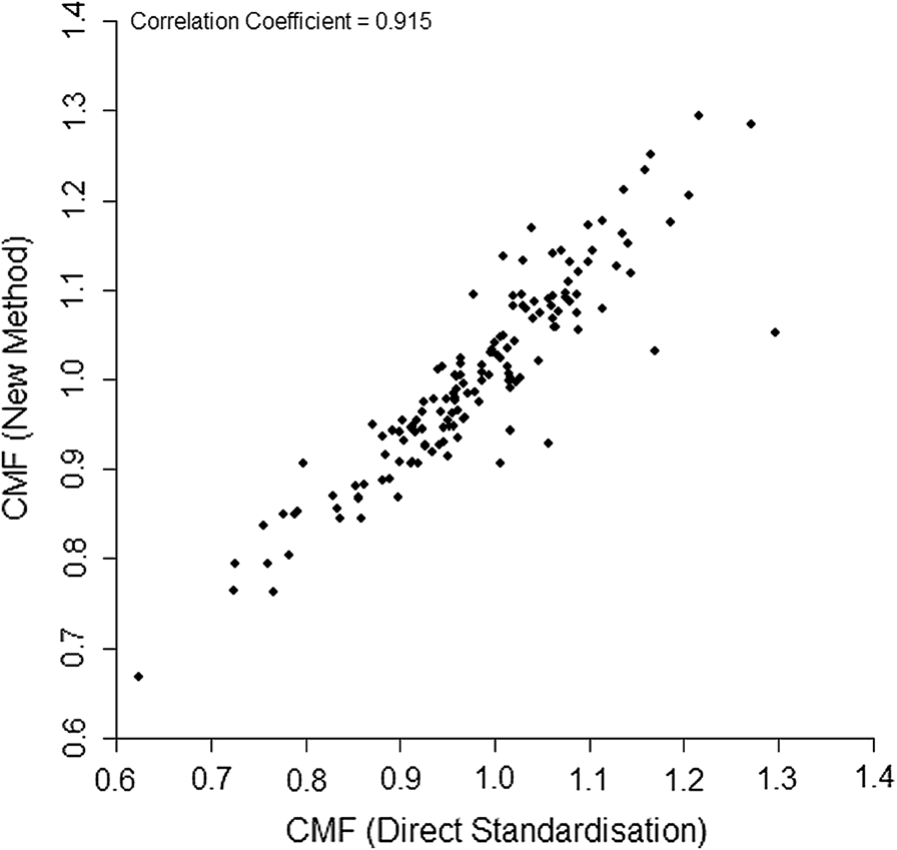
**Scatterplot comparing the directly risk standardised CMF calculated using the new method vs the conventional directly casemix standardised CMF** (**calculated with adjustment of the weights for missing casemix groups**) **in 146 hospitals in England.**

**Figure 3:**
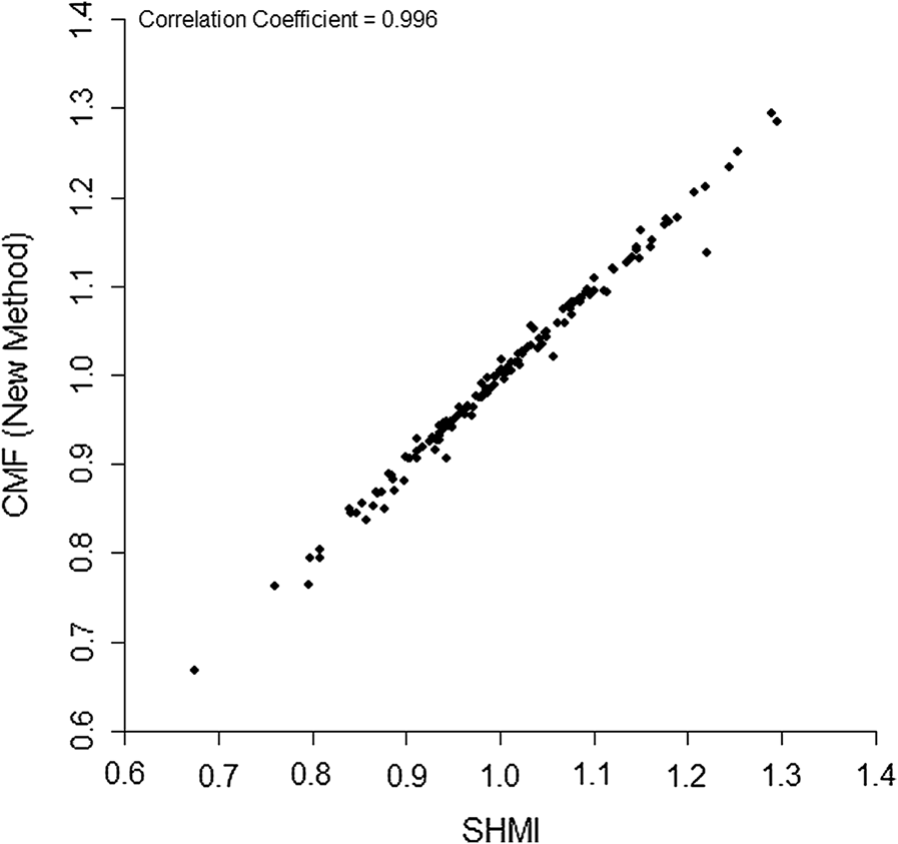
Scatterplot comparing the directly risk standardised CMF calculated using the new method vs the SMR calclulated using the SHMI model in 146 hospitals in England.

Figures 2 and 3 compare the different methods of standardisation of hospital mortality rates in the HES data. Figure 2 shows that there is a difference between the results for conventional direct casemix standardisation and our proposed direct risk standardisation which could have an impact on the assessment of performance. For example, two of the hospitals in the worst eight for poor mortality performance using conventional direct casemix standardisation are not in the worst 40 using our proposed method. However, Figure 3 shows that the new method very closely replicates the SHMI which is an SMR.

**Figure 4:**
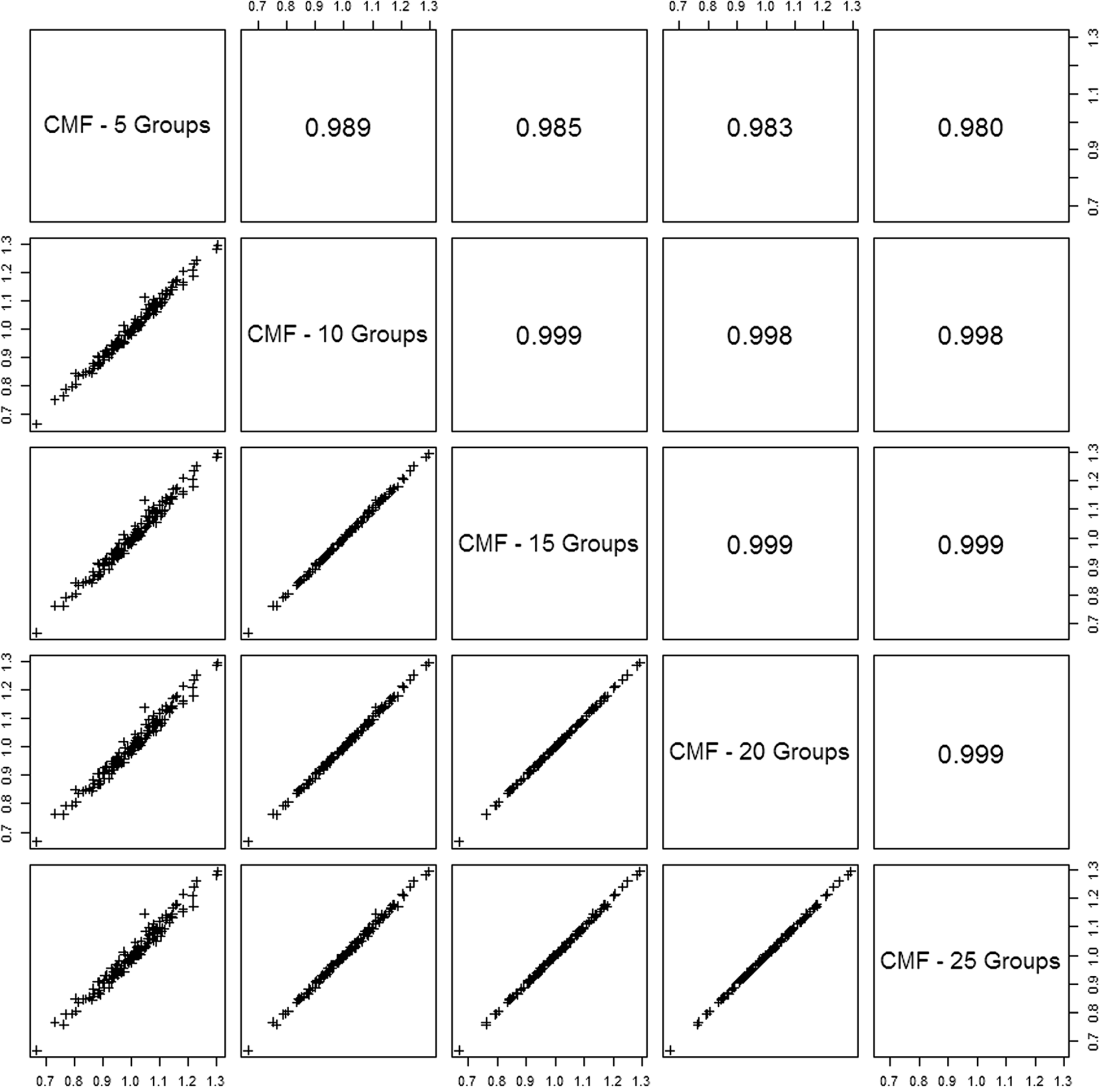
Scatterplots and Spearman rank correlations comparing the directly risk standardised CMF when calculated using different numbers of risk categories.

Figure 4 shows the correlation between the DRS CMFs for the 146 hospitals in the HES data calculated using between 5 and 25 risk categories. It will be seen that there is some discrepancy between the DRS CMFs calculated using 5 categories and the most reliable estimate using 25 categories, with a rank correlation of 0.980. However, when 10 categories are used the correlation increases to 0.998 indicating that in this dataset 10 categories were sufficient to calculate a reliable standardised rate.

### DAVROS data

Table 4 shows the SMRs and the CMF calculated using the proposed DRS method for the nine centres in the DAVROS data. Again it will be seen that the CMF calculated from the directly risk standardised rate and the SMR are very similar. Table 4 also shows the standard errors (SEs) calculated from the observed data using the formulae given in the Appendix, and also calculated by the bootstrap method. It will be seen that the standard errors of the SMR and the CMF are also very similar.

**Table 4:** **Observed values**, **and standard errors** (**SEs**) **and bootstrapped standard errors**, **for the SMR and CMF for nine centres in the DAVROS data**

**Centre**	**SMR**			**CMF**		
	**Observed value**	**SE (theoretical)**	**SE (bootstrap)**	**Observed value**	**SE (theoretical)**	**SE (bootstrap)**
**A**	1.03	0.095	0.076	1.06	0.083	0.077
**B**	1.19	0.084	0.069	1.19	0.076	0.070
**C**	0.92	0.061	0.046	0.90	0.053	0.047
**D**	0.95	0.097	0.083	0.97	0.094	0.089
**E**	0.99	0.109	0.090	1.01	0.099	0.092
**F**	0.84	0.098	0.081	0.84	0.091	0.084
**G**	1.01	0.115	0.101	1.02	0.102	0.101
**H**	0.98	0.131	0.105	0.96	0.114	0.112
**I**	1.08	0.129	0.104	1.08	0.111	0.106

## Discussion

We have illustrated a new method for direct standardisation of event rates that is

• As easy to calculate as the SMR

• Creates an index, the CMF which is similar to the SMR, or a standardised rate

• Can be calculated using continuous covariates

• Unlike the SMR, can be used to compare populations, centres or time periods fairly

• Has an easily estimated standard error that is similar to the SMR

The method converts the complex multi-dimensional casemix to a single dimensional risk distribution, and this can be seen as a development of the methods proposed by Hollis [13]. She proposed that W scores, which are similar to SMRs but are the difference in oberved and expected events rather than their ratio, should be calculated in a few risk categories and then combined using a standard set of weights in order to make fair comparisons between centres with different casemix. Glance [7] proposed the same approach for calculating SMRs in risk categories and then combining these to enable fair comparisons of SMRs between centres. However, rather than calculating SMRs or W scores in risk categories, it is simpler to calculate the actual event rates in each category and then combine them as we have proposed.

We can’t overcome the problem of non-comparability of SMRs or W scores by using direct casemix standardisation of the event rates because of the problem of different patterns of empty casemix groups in different centres occurring for random or organisational reasons. It could be argued that no comparisons should be made between institutions with different patterns of organisational zeros because comparisons between types of institution, such as women’s hospitals, children’s hospitals, mental health hospitals, independent treatment centres only doing elective cases, and general hospitals, are not meaningful [14]. So the real problem is the occurrence of random zeros, and in some cases this could be solved by increasing the size of the dataset, eg taking two years of data, or collapsing the casemix categories, eg taking 10 year age bands rather than 5 year bands. However, in the sorts of models we have been considering with tens of thousands of casemix combinations this may not solve the problem. Furthermore, there would still be the need to omit or categorise continuous covariates.

We have suggested that one approach to get around the problem of empty casemix combinations in conventional direct casemix standardisation might be to re-calculate the weights actually used in each centre so that they always sum to one. Unfortunately this is only a partial solution since it now means that each centre could be using a different set of weights and so, in exactly the same way as for indirect standardisation, comparisons between centres are not fair.

The Directly Risk Standardised CMF is not exact (in the sense of guaranteeing that two centres with identical casemix event rates have identical CMFs) unless all the cases in each of the risk categories have exactly the same risk as each other. This will not usually be true. However, the inaccuracy is related to the number of categories used because with more categories all the cases in a category are more likely to have the same risk as each other. We calculated the effect of using different numbers of categories in the HES data and found negligible differences between using 10 and 25 categories. We therefore suggest that typically about 10 categories should be used. However, if there are some centres with very few cases then this may lead back to the problem that in these centres there may be some risk categories with no cases and direct standardisation methods, including the DRS method, will not work. In this case it may be necessary to use fewer categories. In her example for comparing trauma centres Hollis [13] uses 6 categories for standardising W scores. An alternative would be to omit small centres with empty categories from comparisons since with very few events their standardised rates may be too unreliable for robust comparisons anyway. The alternative of reverting to indirect standardisation using SMRs is not recommended unless the casemix of the centres to be compared has been shown to be similar since studies have shown that if this is not true then there may be substantial biases in the comparison of SMRs [7,15].

In our examples, comparing hospitals with similar casemix and large samples, we found very little difference between the SMRs and the directly risk standardised CMFs. This has been found before [10] though the authors of that study also showed that when casemix differs between hospitals, SMRs vary between hospitals providing the same quality of care. They concluded that direct standardisation was theoretically preferable, but “practically impossible when multiple predictors are included in the casemix adjustment model”. We have shown that, on the contrary, it is possible using risk standardisation.

The example we have used suggests the standard errors of the SMR and Directly Risk Standardised CMF are similar. It remains to be determined whether this is generally true. The bootstrap seems to suggest that the theoretical formulae overestimate the standard error, but the standard errors for the SMR and CMF are similar with both the theoretical values and the bootstrap ones.

The fact that we found little difference between the DRS CMFs and the SMRs, and between their standard errors, points to an important limitation of our study. We do not know to what extent these findings depend on the particular examples we have chosen. We do know that as the casemix differences between centres being compared increase, the biases in SMRs increase, and hence the likely discrepancy from the DRS CMF. But we haven’t quantified these biases, and we don’t know what characteristics determine the relative standard errors of the two methods. Hence we don’t know in what circumstances indirect standardisation should be rejected in favour of the DRS CMF. A large simulation study comparing all methods of standardisation, reflecting real life data with missing values, and looking at outcomes such as the detection of outliers would be necessary.

## Conclusions

In conclusion, it should be reiterated that all methods of standardisation require specification of a 'risk’ model, and the choice of this model is probably more important than the method of standardisation. Nevertheless for a given model it is important that the best method of standardisation should be used and since direct standardisation using the DRS method is as straightforward as using the SMR and overcomes the problem of the non-comparability of SMRs, it should be preferred when the centres being compared may have different casemix profiles and tables of comparative performance using standardised measures are being constructed.

## Abbreviations

CMF: Comparative mortality figure; DRS: Direct risk standardisation; DSR: Directly standardised rate; HES: Hospital episode statistics; SHMI: Summary hospital mortality index; SMR: Standardised mortality ratio.

## Competing interests

The authors declare that they have no competing interests.

## Authors’ contributions

JN conceived the idea, analysed the DAVROS data, and drafted the manuscript. RJ helped conceive the idea, analysed the HES data, and helped revise the manuscript. MJC helped conceive the idea, and revise the manuscript. All authors read and approved the final manuscript.

## Pre-publication history

The pre-publication history for this paper can be accessed here:

http://www.biomedcentral.com/1471-2288/13/133/prepub

## Supplementary Material

Additional file 1 **Appendix.** It contains technical formulae for the calculation of the DRS rates, SMR, and their standard errors. Click here for file
