# Supplementary material for: Direct risk standardisation: a new method for comparing casemix adjusted event rates using complex models
Source: BMC Med Res Methodol. 2013 Oct 29;13:133. doi: 10.1186/1471-2288-13-133 (PMC3870993; doi:10.1186/1471-2288-13-133)
Supplement: Additional file 1 — Appendix. It contains technical formulae for the calculation of the DRS rates, SMR, and their standard errors. [file 1471-2288-13-133-S1.docx]

**Appendix**

Directly Risk Standardised Rate (DRSR)

The DRSR is the sum of the weighted, risk category specific event rates

DRSR_k_  $=$ $\sum_{j=1}^{J} w_{j}\frac{d_{jk}}{n_{jk}}$ (1)

where n_jk_ is the number of cases (eg patients) in the j^th^ risk category in centre k, d_jk_ is the number of events (e.g. deaths) in the j^th^ group, and w_j_ is the weight for the j^th^ group. These weights are usually taken from a ‘standard’ population, or are the proportion of the cases that are in the jth category in all centres together, ie

$$w_{j}=\frac{\sum_{k=1}^{K} n_{jk}}{\sum_{k=1}^{K} \sum_{j=1}^{J} n_{jk}}$$

Assuming the number of cases in the j^th^ risk category, nj, is fixed, and the number of events, dj, is binomially distributed, then the standard error of the DRSR is given by

SE(DRSR_k_) = $\sqrt{\sum_{j=1}^{J} w_{j}^{2}\frac{d_{jk}\left( n_{jk}-d_{jk} \right)}{n_{jk}^{3}}}$ (2)

In fact, in the DRSR with the methods we have used, we have chosen the categories to have a fixed number of events, and it is the number of cases in each category which is variable. The number of cases in each category can be considered to follow a negative binomial distribution in this formulation. However, as you might expect the resulting estimate of the standard error is the same as with the binomial formulation given above which can be shown by noting that from (1), since d_jk_ is considered fixed

$Var\left( {DRSR}_{k} \right)=\sum_{j=1}^{J} w_{j}^{2}d_{jk}^{2}Var\left( \frac{1}{n_{jk}} \right)$.

Now for a random variable y, $Var\left( \frac{1}{y} \right)\simeq\frac{Var(y)}{{E(y)}^{4}}$, and for a negative binomial distribution with a fixed number of failures d and probability of failure p then, $E\left( n \right)=\frac{d}{p}$ where n is the total number of cases or trials, i.e. successes + failures, and $Var\left( n \right)=\frac{\left( 1-p \right)d}{p^{2}}$. [9]

So $Var\left( {DRSR}_{k} \right)=\sum_{j=1}^{J} w_{j}^{2}\frac{(1-p_{jk})p_{jk}^{2}}{d_{jk}}$ and letting $p_{jk}=\frac{d_{jk}}{n_{jk}}$ ,

$Var\left( {DRSR}_{k} \right)=\sum_{j=1}^{J} w_{j}^{2}\frac{d_{jk}(n_{jk}-d_{jk})}{n_{jk}^{3}}$ as in (2).

Comparative Mortality Figure (CMF)

The CMF is the ratio of the directly standardised rate for the cohort to the standard population rate. The equation for the CMF can be expressed as

CMF_k_ = DRSR_k_ / standard population rate

$${CMF}_{k}=\frac{{DRSR}_{k}}{\sum_{j=1}^{J} w_{j}\frac{D_{j}}{N_{j}}}$$

Where D_j_ is the number of deaths in j^th^ group of the standard population and N_j_ is the number of patients in the j^th^ group of the standard population. When the weights w_j_ are just the proportion of the standard population in the jth category, the denominator equals the aggregate population death rate, that is the ratio of total deaths (D) to total population (N).

The approximate standard error of the CMF is then given by

$$\text{SE}\left( {CMF}_{k} \right)\text{=}\frac{SE\left( {DRSR}_{k} \right)}{\frac{D}{N}}$$

Due to the skewed distribution of the CMF it may be preferred to transform it to the log scale. The approximate standard error for the transformed CMF is

$$\text{SE}\left( \log\text{CMF} \right)\text{=}\frac{\text{SE}\left( \text{CMF} \right)}{\text{CMF}}$$

Standardised Mortality Ratio

The SMR is the ratio of the observed number of deaths in a study population to the expected number of deaths:

$$\text{SMR=}\frac{\text{Observed deaths (in study population)}}{\text{Expected deaths (in study population)}}=\frac{O}{E}$$

The standard error of the SMR is approximately

$$SE\left( SMR \right)=\frac{\surd O}{E}$$

As with the CMF it may be preferable to use the log transformed SMR to account for its skewed distribution. The approximate standard error for the transformed SMR is

$$\text{SE}\left( \log\text{SMR} \right)\text{=}\frac{\text{SE}\left( \text{SMR} \right)}{\text{SMR}}\text{=}\frac{\text{1}}{\text{√O}}$$

Reference

9. Hilbe JM(2011) Negative Binomial Regression 2^nd^ Ed Cambridge: Cambridge University Press
